# Exfoliated Graphite as a Solid Sorbent in Ultrasound-Assisted Dispersive Micro-Solid-Phase Extraction for Determination of Chromium and Vanadium in Herbs

**DOI:** 10.3390/foods14234075

**Published:** 2025-11-27

**Authors:** Małgorzata Osińska, Piotr Krawczyk, Magdalena Krawczyk-Coda

**Affiliations:** Faculty of Chemical Technology, Poznan University of Technology, Berdychowo 4, 60-965 Poznań, Poland; malgorzata.osinska@put.poznan.pl (M.O.); piotr.krawczyk@put.poznan.pl (P.K.)

**Keywords:** high-resolution continuum source GFAAS, ultrasound-assisted dispersive micro solid-phase extraction, exfoliated graphite, chromium, vanadium, herbs

## Abstract

In this research, a preconcentration procedure was developed for the sequential determination of chromium and vanadium using high-resolution continuum source graphite furnace atomic absorption spectrometry (HR-CS GFAAS). Due to low concentrations, chromium and vanadium were determined following preconcentration onto exfoliated graphite using ultrasound-assisted dispersive micro-solid-phase extraction (US DMSPE). The experimental parameters, including pH of the sample solution, the amount of exfoliated graphite, extraction time, elution conditions, as well as the main parameters of HR-CS GFAAS, were investigated. The calculated limits of detection for Cr and V were 0.003 µg g^−1^ and 0.006 µg g^−1^, respectively. The preconcentration factors obtained for Cr and V were 28 and 34, respectively. The RSD ranged from 0.3% to 3.4% for Cr and from 0.9% to 4.6% for V. The accuracy of this method was validated by analyses of INCT-MP4-2 (Mixed Polish Herbs) certified reference material. The measured chromium and vanadium contents were in satisfactory agreement with the certified values according to the *t*-test for a 95% confidence level. The proposed method was successfully applied for the determination of both elements in herbs such as hawthorn flower, hawthorn fruit, motherwort, white mulberry leaf, common milkweed, mistletoe, valerian root, and horse chestnut bark.

## 1. Introduction

Lifestyle diseases develop slowly, often over many years, as a result of the way people live in modern society. The most common lifestyle diseases are cardiovascular diseases (high blood pressure, heart attack, and stroke), obesity and overweight, and type 2 diabetes [1,2].

In recent years, interest in natural medicine and herbal therapy has grown significantly. Numerous scientific studies confirm the effectiveness of certain herbs in supporting health, reducing symptoms, and preventing disease. Although herbs should not completely replace modern medical treatment, they can play a significant role in improving overall health and quality of life. Herbs are rich in biologically active compounds, including flavonoids, alkaloids, essential oils, tannins, and minerals. By using herbs, patients can often reduce the side effects of chemical drugs and strengthen their natural resistance. Herbs usually work gently and gradually, improving overall well-being. However, some herbs can interact with prescription drugs, and incorrect dosage or poor-quality herbs can be harmful. Additionally, herbal therapy requires regular use and patience, as results appear slowly [3,4,5,6].

White mulberry leaves are most often used to treat type 2 diabetes due to their impact on sugar metabolism, which may support weight loss by helping maintain stable blood sugar levels and reducing cravings for sweets [7,8,9]. Motherwort [10] and hawthorn berry [11] promote digestive health and can alleviate issues such as flatulence and indigestion. Hawthorn flower and fruit are known for their cardioprotective properties [12]. They help improve heart function by strengthening the heart muscle and supporting blood circulation. Motherwort supports heart health by relieving symptoms such as palpitations and mild cases of tachycardia. It also helps improve circulation and acts as an aid in regulating blood pressure [13]. White mulberry contains antioxidants that can support heart health by reducing oxidative stress and improving blood lipid profiles [14].

Chromium (Cr) is a component of the glucose tolerance factor (GTF), which enhances the action of insulin, the hormone responsible for regulating blood glucose levels. This element also influences the metabolism of carbohydrates, fats, and proteins, playing a crucial role in maintaining stable energy levels and body weight [15,16]. Vanadium (V) is not officially recognised as an essential nutrient for humans, but research suggests that it can influence insulin activity and glucose regulation [17,18].

The determination of Cr and V in herbs can be challenging due to their low concentrations and matrix interferences. As a result, the direct determination of these elements using graphite furnace atomic absorption spectrometry (GFAAS) often proves difficult. To overcome these challenges, the use of nanomaterials for preconcentrating analytes and separating them from the matrix is often necessary [19].

Exfoliated graphite (EGIC) refers to graphite in which a significant portion of the carbon layers has been separated. This separation process is called exfoliation and can involve chemical, mechanical, or thermal methods. EGIC exhibits high adsorption capacity, selectivity, and reusability. Additionally, its production can be both eco-friendly and cost-effective [20].

Dispersive micro-solid-phase extraction (DMSPE) using various nanomaterials as solid sorbents is gaining popularity due to its numerous advantages, including the immediate interaction between metal ions and the adsorbent, as well as the shorter time required for sample preparation compared to classical solid-phase extraction (SPE) [21,22].

The literature includes several papers related to the preconcentration of Cr and V on solid sorbents before the determination by graphite furnace atomic absorption spectrometry. Chromium has been successfully preconcentrated on magnetic ferrite [23], graft copolymer composed of polystyrene and polylinoleic acid with the sodium iminodiacetate salt [24], tailor-made polymer nanoparticles [25], and graphene oxide nanoparticles [26] using DMSPE. The GF AAS technique has been applied for vanadium preconcentration after cloud point extraction based on the formation of a ternary complex between vanadium, 2-(2’-thiazolylazo)-p-cresol, and ascorbic acid [27], eutectic solvent extraction [28], magnetic stirrer-induced dispersive ionic-liquid microextraction [29], and extraction induced by emulsion breaking [30].

This project aimed to develop an analytical procedure for determining Cr and V in herbs and their infusions using high-resolution continuum source graphite furnace atomic absorption spectrometry (HR-CS GFAAS). Due to the low concentrations of these analytes, Cr and V were determined after ultrasound-assisted dispersive micro-solid-phase extraction (UA DMSPE) with exfoliated graphite serving as the solid sorbent. This preconcentration technique facilitates immediate interaction between the analyte and the adsorbent, thereby reducing sample preparation time. To our knowledge, this is the first methodology presented in the literature for the sequential determination of Cr and V in herbs following preconcentration on exfoliated graphite.

## 2. Materials and Methods

### 2.1. Reagents

Compressed argon of UHP 5.5 purity obtained from Air Products (Poland) was employed as an inert gas without further purification. Working standard solutions of Cr and V were prepared from a 1000 mg L^−1^ atomic absorption standard solution (Merck, Darmstadt, Germany). Palladium (Pd(NO_3_)_2_) and magnesium (Mg(NO_3_)_2_) modifier stock solutions (10.0 ± 0.2 g L^−1^ for each element) were obtained from Merck. For the sample pH adjustment, 65% (*v*/*v*) HNO_3_ and 30% (*v*/*v*) NaOH of the highest quality (Suprapur, Merck, Darmstadt, Germany) after proper dilution were used. Nitric acid 65% (*v*/*v*) and hydrogen peroxide 30% (*v*/*v*) of the highest quality (Suprapur, Merck, Darmstadt, Germany) were used for sample digestion. Nitric acid at a concentration of 18 mol L^−1^ (purity 100%, Merck, Darmstadt, Germany) was used during galvanostatic oxidation of graphite. Deionised and doubly distilled water (quartz apparatus, Bi18, Heraeus, Hanau, Germany) was used throughout the experiments. The resistivity of the water was 18 MΩ cm.

### 2.2. Certified Reference Materials and Herbs

The accuracy of the analytical procedure described in this study was assessed using the INCT-MP4-2 (Mixed Polish Herbs) certified reference material, provided by the Institute of Nuclear Chemistry and Technology in Warsaw, Poland.

In this study, eight herbs were analysed: hawthorn flower, hawthorn fruit, motherwort, white mulberry leaf, common milkweed, mistletoe, valerian root, and horse chestnut bark. All herbs were sourced from organic farms in the Podlasie region of eastern Poland and were purchased from a local herbal store. Three packages of each herb were purchased. They came from different batches of the product. Before digestion, each sample was dried, mixed, manually ground in a ceramic mortar to ensure homogeneity, and sieved through a smaller than 2 mm sieve.

### 2.3. Exfoliated Graphite Preparation and Characterisation

The investigated material, exfoliated graphite, was obtained by a two-part synthesis, which includes HNO_3_ intercalation into the pristine graphite followed by thermal exfoliation of the beforehand yielded graphite intercalation compound with nitric acid (HNO_3_-GIC). The process of intercalation was performed by the electrochemical method using a galvanostatic technique. The choice of intercalation method was dictated by the advantages of electrochemical methods over chemical ones. Among the most important advantages of electrochemical intercalation are advanced control of the process and reduced reagent consumption, which results in fewer waste products that would need to be recycled.

A constant current of 2.5 mA was passed through the electrode made of graphite flakes (purity 99.5%, flake size ranged between 170 and 283 μm) until the electrode reached a potential of 1.1 V. Galvanostatic oxidation of natural graphite was performed in an aqueous solution of 18 mol L^−1^ HNO_3_. An electrochemical intercalation was carried out in a three-electrode system, in which platinum wire (purity 99.9%, 1 mm diameter) served as a counter electrode, whereas Hg/Hg_2_SO_4_/1 mol L^−1^ H_2_SO_4_ played the role of reference electrode. An electrochemical intercalation was performed using a potentiostat–galvanostat PGSTAT30 AutoLab (EcoChemie B.V., Ultrecht, The Netherlands). The synthesised graphite intercalation compound with nitric acid was washed with distilled water until the filtrate became neutral, and after that, dried in air at ambient temperature. The final product, HNO_3_-GIC, was composed mainly of the stage 2 phase, which means that two neighbouring layers of intercalate were separated by the two graphene layers. Details of electrochemical intercalation of nitric acid into the graphite flakes concerning the electrode preparation as well as the characterisation of the yielded product can be found in our previous studies [31,32].

The as-prepared graphite compounds underwent thermal treatment for 4 min at 800 °C in an air atmosphere to conduct the process of exfoliation. The mentioned process was conducted at ambient temperature in a chamber furnace (Lenton, Hope Valley, UK). The sample acquired after the exfoliation process was called EGIC.

Morphological properties of carbon electrodes were studied by scanning electron microscopy (SEM) (Hitachi S-3400N, Hitachi, Sydney, Australia) coupled with the energy-dispersive spectrometer (EDS) (Thermo Electron Corp., Madison, WI, USA, model No. 4481B-1UES-SN with the NSS v. 3.3 Spectral Imaging System software).

The porosity of the samples was characterised by means of nitrogen adsorption–desorption isotherms at −195.5 °C at a relative pressure in the range of 0.06 to 0.30 in an ASAP2020 V3.01 H–Micromeritics device. The BET specific surface area (SBET) of sample was determined from the nitrogen adsorption branch by applying the Brunauer–Emmet–Teller equation.

Fourier transform infrared spectroscopy (FTIR) was applied to evaluate the surface chemical structure of the obtained carbon. JASCO (model 6700, type A) was employed. The data were recorded between 4000 and 400 cm^−1^ over 100 scans at a resolution of 4 cm^−1^.

### 2.4. Microwave-Assisted High-Pressure Teflon Bomb Digestion

Certified reference material and herbs were digested in a focused microwave sample preparation system (UniClever, Plazmatronika, Wrocław, Poland) operating at 2450 MHz and 300 W maximum output. The system was controlled by a computer, which allowed for the continuous monitoring of temperature, pressure, and microwave power. The samples were digested in a high-pressure TFM-PTFE vessel with a capacity of 110 mL, a maximum allowed pressure of 100 atm, and a maximum temperature of 300 °C.

Approximately 300 mg of powdered certified reference material or herbs was placed in the TFM-PTFE vessel of the microwave system and moistened with 1 mL of 30% H_2_O_2_. Next, 5 mL of 65% HNO_3_ was added. The samples were then heated for 20 min at 300 W. After the microwave-assisted digestion, the clear solutions were transferred into 20 mL calibrated flasks and diluted to a specified volume with high-purity water. Before further analysis, the samples were appropriately diluted based on the concentration levels of Cr and V. A corresponding blank was also prepared following the same microwave-assisted digestion procedure described above.

### 2.5. Preparation of Herbal Infusions

During the research, Cr and V were also determined in herbal infusions. To prepare the infusions, one tablespoon of dried herbs was combined with 250 mL of boiling water and allowed to steep, covered, for 15 min. After steeping, the infusions were filtered using a Cameo syringe filter with a polytetrafluoroethylene membrane and a pore size of approximately 0.22 mm (GE Water & Process Technologies, Feasterville Trevose, PA, USA). The filtered infusions were then stored at 4 °C in polyethylene flasks for a maximum of three days.

### 2.6. Preconcentration and AAS Determination Procedures

Before extraction, the pH of the samples was adjusted to 6.5 using a pH metre (pH 211 Microprocessor, Hanna Instruments, Kehl, Germany) supplied with a glass-combined electrode. Approximately 5 mg of EGIC was weighed using an M2P microanalytical balance (Sartorius, Gottingen, Germany) and added to 10 mL of the sample solution. The preconcentration of Cr and V was conducted using ultrasound-assisted dispersive micro-solid-phase extraction (UA DMSPE) for 10 s with an ultrasound probe equipped with a 2 mm titanium microtip (Sonopuls HD 70, 70 W, 20 kHz, Bandelin, Berlin, Germany).

To separate the phases, the resulting suspension was centrifuged for 2 min at 4500 rpm using a centrifuge (model EBA 20, Hettich, Germany). The analytes adsorbed onto the material were then eluted (re-extracted) with 250 μL of 2 mol L^−1^ HNO_3_. The elution was performed for 10 s using an ultrasonication bath, followed by centrifugation for 1 min at 4500 rpm. Finally, 20 μL of the eluent and 5 μL of a modifier solution Mg(NO_3_)_2_ at a concentration of 3 mg mL^−1^ were injected into a graphite furnace for analysis.

During the experiments, a high-resolution atomic absorption spectrometer equipped with a continuum radiation source as a 300 W xenon short-arc lamp (ContrAA 700, Analytik Jena, Jena, Germany) was used. As an atomiser, a graphite furnace with a pyrolytically coated graphite tube with a platform was employed.

## 3. Results and Discussion

### 3.1. Characterisation of Synthetised Material

**Figure 1:**
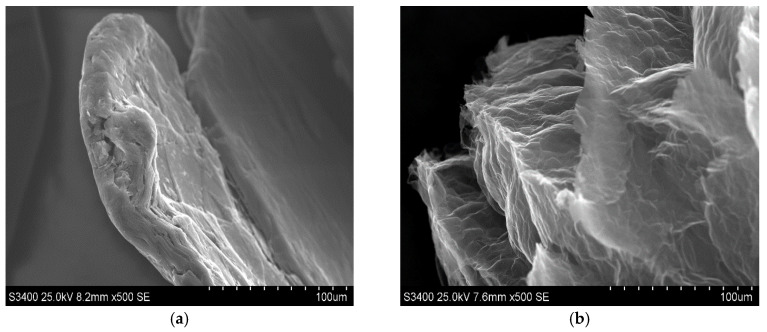

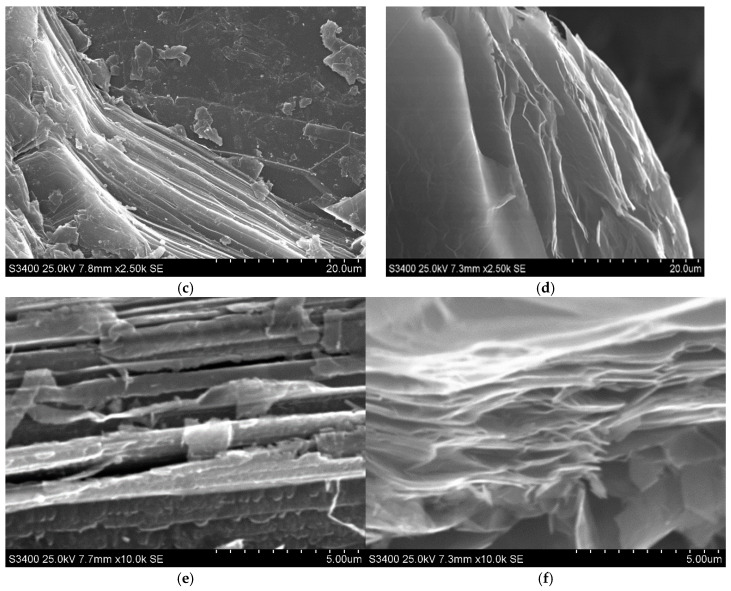
SEM images of original graphite (**a**,**c**,**e**) and exfoliated graphite (EGIC) (**b**,**d**,**f**) recorded under different magnifications.

The morphological properties of the synthetised material were estimated on the basis of SEM images recorded for the exfoliated graphite, which are displayed in Figure 1b,d,f, to facilitate the observation of morphology caused by the intercalation followed by thermal treatment of graphite. Figure 1 also contains images of pristine graphite (Figure 1a,c,e).

**Figure 2:**
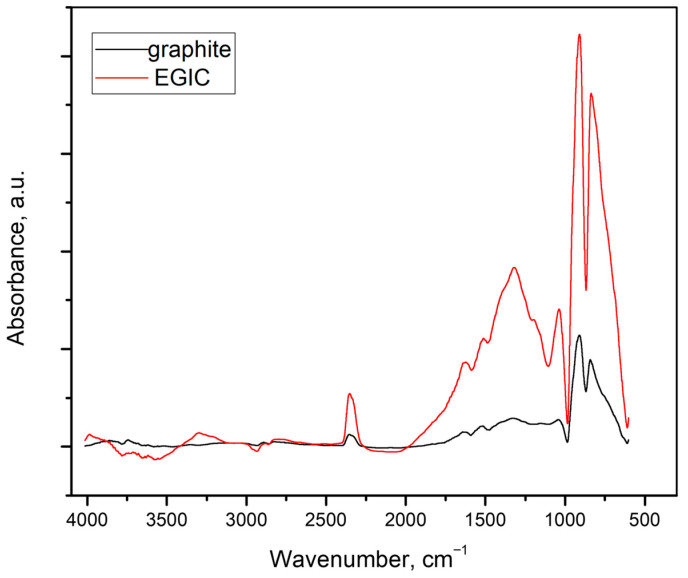
FTIR spectra of original graphite and exfoliated graphite (EGIC).

As one can see, SEM images for pristine graphite (Figure 1a,c,e) exhibit typical shapes for graphite flakes, consisting of flat surfaces with some amount of surface defects mainly occurring on the edges of graphene layers. Graphene layers are arranged one beneath the other, forming a kind of stack with visible gaps between the layers, into which the intercalate is introduced during the intercalation process. As this process progresses, the intercalate diffuses into the spaces between the graphene layers, occupying increasingly deeper positions. The above-described morphological effects are caused by the intercalation process during which the intercalate locates between the graphene layers, which is commonly accompanied by the more or less significant damage of graphite matrix. From the observation of SEM images of exfoliated graphite (Figure 1b,d,f), it is obvious that the process of exfoliation leads to much more significant changes in morphology, which is illustrated by the huge increment in flake volume. The rapid evolution of products of thermal decomposition of intercalate results in an increase in the volume of the graphite matrix along the c-axis. The stage of morphological modification of graphene layers is strongly determined by the type of exfoliation agent as well as the type of graphite intercalation compounds subjected to exfoliation. As one can see, the significant increase in graphite volume is accompanied by the increase in concentration of edges and surface defects, which can play the role of surface centres. The above-mentioned information seems to be very important from the practical point of view because it is revealed that exfoliated graphite exhibits properties that allow its practical application in adsorption processes. Additionally, taking into account the oxidative character of the conducted exfoliation, it can be assumed that during the thermal treatment of HNO_3_-GIC, the modification of the chemical composition of the graphite surface occurs, leading to the formation of surface functionalities. The edges and surface defects, which are so numerous on the surface of exfoliated graphite, are particularly favourable for the formation of these groups. In order to confirm the above-mentioned statement, the FTIR analysis of the examined exfoliated graphite was performed, and the recorded spectrum is shown in Figure 2. To enable interpretation, the spectrum of pristine graphite is also involved in the regarded figure.

From the comparison of the spectra presented in Figure 2, it is clear that the surface of exfoliated graphite is much better in surface functional groups than the surface of pristine graphite. The intensity of the observed signals is significantly higher for exfoliated graphite. On the basis of this preliminary observation, it can be assumed that the thermal treatment of graphite flakes filled with nitrogen-containing intercalate performed under oxidative conditions, given by an air atmosphere, resulted in the formation of a wide range of surface functionalities which involve oxygen but also nitrogen atoms. FTIR spectra are composed of two main vibration bands, the first of which extends over wavelengths between 986 and 611 cm^−1^, whereas the second one from 2011 to 986 cm^−1^. Within the first range, there are two intensive signals which probably arise from the exfoliation and can be aligned to C-O and C-H bonds [33,34,35].

The latter band is much broader and involves more signals of different intensities and origins. It is plausible that both peak at a wavelength of 1320 cm^−1^, and the small signal positioned on the left shoulder of this peak (1378 cm^−1^) can be regarded as a residual of intercalate because they may be assigned to NO^3-^ and C-O-N [33,36]. Other signals pertaining to the considered bands, 1514, 1628, and 1037 cm^−1^, arise from graphitic skeleton (C-C), surface functionalities which contain heteroatoms of oxygen (C-O and C-O in COOH groups), respectively [33,34].

The FTIR spectra clearly show that the surface of exfoliated graphite is significantly richer in surface functional groups than the surface of pristine graphite. However, it should be noted that FTIR spectra do not allow for quantitative estimates. This inaccuracy can be compensated for by the results of the EDS analysis, which clearly show that, on average, the surface of exfoliated graphite consists of 95.5% carbon and 4.5% oxygen, while the surface of the pristine graphite consists of 99.8% carbon.

The specific surface area of exfoliated graphite reached 28.94 m^2^/g. This is the result of thermal exfoliation carried out under shock conditions in an air atmosphere. It is worth noting that the specific surface area of the initial material (graphite used for the intercalation of HNO_3_) was only 0.37 m^2^/g. This surface development indicates that mesopores with a small proportion of micropores dominate the porous structure of exfoliated graphite. This may indicate a wide range of materials that can be sorbed on the surface of exfoliated graphite, taking into account the size of the sorbed particles.

Summarising, it can be pointed out that exfoliated graphite, being the product of thermal exfoliation of graphite intercalation compound with nitric acid, exhibits very interesting properties caused by the presence of an increased amount of edges and surface defects, which are, in some part, enriched with a variety of surface functionalities.

### 3.2. Optimisation of HR-CS GFAAS Detection

The optimised operating parameters of the spectrometer are presented in Table 1. Chromium and vanadium were determined at their most sensitive wavelengths, specifically 357.8687 nm for Cr and 318.3982 nm for V. The transient absorbance signals were integrated, and both peak height and peak area signals were recorded. It was found that the precision of the peak area measurement was superior to that of the peak height measurement; therefore, peak areas of the absorbance signals were utilised for calculations. An analytical blank was employed throughout the procedure, and the mean blank values (0.6 ng for Cr and 1.0 ng for V) were subtracted from the sample values. Calibration was performed using the standard calibration technique, with standards prepared according to the proposed extraction procedure.

Pyrolysis temperature is a critical parameter in the temperature programme for determining Cr and V using HR-CS GFAAS. The temperature programme was optimised for standard solutions containing 25 µg L^−1^ of both Cr and V. The conditions were adjusted to continuously search for the optimal balance of stabilising ability, sensitivity, and precision. The influence of pyrolysis temperature on absorbance was investigated within the ranges of 1200–1500 °C for Cr and 1100–1400 °C for V, using a modifier. Maximum absorbance was achieved at a pyrolysis temperature of 1400 °C for Cr and 1200 °C for V. After optimising pyrolysis conditions, the effect of atomisation temperature on the analytical signals of Cr and V was examined within the ranges of 2300–2600 °C for Cr and 2500–2800 °C for V. The optimum pyrolysis temperatures established were 1400 °C for Cr and 1200 °C for V. The maximum absorbance for atomisation was reached at 2400 °C for Cr and 2800 °C for V. The atomisation temperature for vanadium was not increased above 2800 °C due to the temperature resistance of the graphite tube. During the research, the effects of modifiers, specifically Pd(NO_3_)_2_ and Mg(NO_3_)_2_, on the analytical signals were examined to achieve optimal sensitivity and precision. For chromium, no significant difference in absorbance was observed in the presence of modifiers. However, in the case of vanadium, the best analytical signals were obtained using a magnesium modifier at a concentration of 3 mg mL^−1^. Complete vaporisation and atomisation of the elements were achieved in the graphite tube, with minimal influence from the matrix. The temperature programme used for the determination of Cr and V in certified reference material and herbs is detailed in Table 1.

### 3.3. Effect of Sample pH

**Figure 3:**
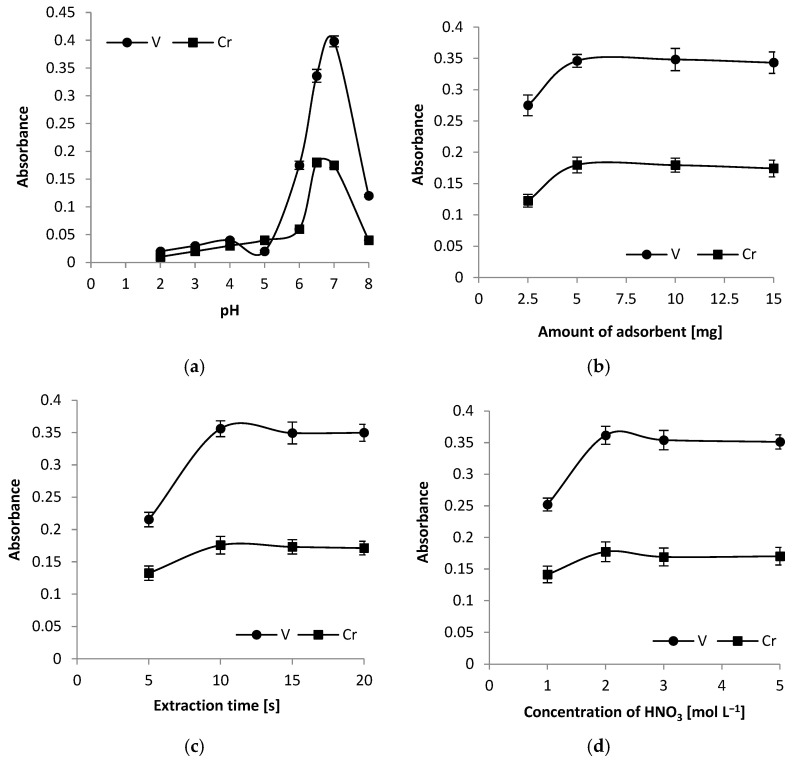
Optimisation of experimental conditions for ultrasound-assisted dispersive micro-solid-phase extraction (UA DMSPE) using exfoliated graphite as a sorbent for a 10 mL sample at a concentration of 10 µg L^−1^ for Cr and V: (**a**) Influence of pH. Conditions: centrifugation time, 3 min, ultrasonication time, 25 s, amount of adsorbent, 5 mg, and eluent concentration, 3 mol L^−1^; (**b**) Influence of adsorbent amount. Conditions: centrifugation time, 3 min, ultrasonication time, 25 s, eluent concentration, 3 mol L^−1^, and sample pH, 6.5; (**c**) Influence of extraction time. Conditions: centrifugation time, 3 min, amount of adsorbent, 5 mg, eluent concentration, 3 mol L^−1^, and sample pH, 6.5; (**d**) Influence of eluent concentration. Conditions: centrifugation time, 3 min, ultrasonication time, 20 s, amount of adsorbent, 5 mg, and sample pH, 6.5. Each optimisation step was replicated three times, and the results were consistent across all trials. The error bar is the standard deviation (n = 5).

Sample pH plays an important role in the adsorption of Cr and V onto exfoliated graphite and was optimised from 2.0 to 8.0. The results are presented in Figure 3a. The point of zero charge (PZC) for exfoliated graphite can vary depending on its specific properties and how it was processed, but studies report values from 4.1 to nearly 7.8 [37]. The analysis of the graph indicates that the optimal pH value is 6.5 for chromium and 7 for vanadium. Therefore, 6.5 was chosen as the most suitable compromised pH value for both elements. At this pH, there are favourable conditions for the adsorption of both elements onto exfoliated graphite, which enhances extraction efficiency. Adsorption of cations increases with increasing pH. The low adsorption of Cr and V ions on exfoliated graphite at lower pH is due to strong H^+^ ion competition for the available exchange sites. At a pH of approximately 6.0, chromium adsorbs as Cr^2+^ ions, while vanadium adsorbs as VOH^+^ ions [38,39]. Retention of Cr and V ions by exfoliated graphite occurs through two primary mechanisms: physical adsorption and chemical adsorption. Physical adsorption involves the attraction of these ions to the graphite surface through weak Van der Waals forces. This process is reversible, allowing the ions to be easily desorbed. Due to the exfoliated graphite structure, adsorption can also occur on the edges, surface defects, and between the layers of graphite flakes. In contrast, chemical adsorption entails the formation of stronger chemical bonds between the ions and the functional groups (e.g., -CO) present on the graphite surface. This form of adsorption is more stable, enhancing both the efficiency of ion retention and the durability of their adsorption to the sorbent. Both mechanisms contribute to the effectiveness of exfoliated graphite as a sorbent in analytical processes.

### 3.4. Effect of Exfoliated Graphite Amount

To obtain the highest analytical signals in the DMSPE procedure, it is essential to use an appropriate amount of adsorbent. In this study, the optimal quantity of exfoliated graphite needed for the quantitative enrichment of Cr and V was investigated by varying the amount of adsorbent from 2.5 to 15 mg per 10 mL sample. The results showed that absorbance increased with the amount of exfoliated graphite in the range of 2.5 to 5 mg (as shown in Figure 3b). This increase can be attributed to a higher number of active sites on the adsorbent’s surface available for binding metal ions. At 5 mg of exfoliated graphite, absorbance reached its maximum. Beyond this point, from 5 to 15 mg, the absorbance plateaued, indicating that the number of active sites was sufficient to bind all the Cr and V ions present in the samples. Therefore, a quantity of 5 mg of exfoliated graphite was selected for further experiments in this study.

### 3.5. Effect of Extraction Time

The impact of ultrasonication time on the absorbance of analytes was investigated over a range of 5 to 20 s. It was found that shorter extraction times resulted in increased absorbance as the ultrasonication time extended. This trend can be explained by the reduced contact time between the sample solution and the adsorbent. At 10 s of ultrasonication, the absorbance of the analytes reached its maximum level. Beyond this point, between 10 and 20 s, the absorbance remained relatively constant, indicating that a plateau had been reached. In the DMSPE procedure, the ultrasonication time not only affects the absorbance of the analytes but also helps manage the overall duration of the analysis. Consequently, an ultrasonication time of 10 s was chosen for further analysis. The results are presented in Figure 3c.

### 3.6. Optimisation of Re-Extraction Conditions

In this study, the analysis of liquid samples after re-extraction was conducted. To ensure effective elution of the analytes before their injection into the graphite tube, nitric acid was selected as the eluent. The minimum volume of acid required to wet the adsorbent was 250 µL, and this volume was used in subsequent experiments. The efficiency of the elution was evaluated for eluent concentrations ranging from 1 to 5 mol L^−1^ (Figure 3d). It was observed that with a low eluent concentration of 1 mol L^−1^ HNO_3_, the absorbance increased. When the eluent concentration rose to 2 mol L^−1^, the absorbance of the analytes reached its maximum value. Further increases in eluent concentration (above 2 mol L^−1^) resulted in stable absorbance levels for Cr and V. Consequently, an eluent concentration of 2 mol L^−1^ was selected for the remaining experiments.

### 3.7. Adsorption Capacity

To determine the adsorption capacity of exfoliated graphite, 150 mg of the adsorbent was added to 10 mL of a sample solution containing 150 mg L^−1^ of Cr and V. The experiment was conducted at a pH of 6.5. After sonication for 20 s, the mixture was centrifuged for 3 min at 4500 rpm, and the adsorbed analytes were eluted using previously optimised conditions. The concentration of the remaining Cr and V ions in the aqueous phase was measured using the HR-CS GFAAS technique. The adsorption capacity (qt) was calculated using the equation qt = V(C_0_ − C_t_)/W, where C_0_ and C_t_ are the element concentrations before and after adsorption (in mg L^−1^), V is the volume of the sample solution (in litres), and W is the mass of the adsorbent (in grams) [40]. The calculated adsorption capacities were found to be 28.9 ± 0.4 mg g^−1^ for Cr and 60.8 ± 1.3 mg g^−1^ for V.

### 3.8. Evaluation of Adsorbent Reusability

The number of times exfoliated graphite can be reused for analytical purposes depends on several factors, including the type of analyte, the sample matrix, the required purity, and the method of regeneration. To assess the reusability of exfoliated graphene, the adsorbent, after the elution of the analytes, was regenerated by shaking it in a laboratory shaker for 5 min in the presence of HNO_3_ at a concentration of 1 mol L^−1^. The adsorbent was then separated from the acidic solution using a paper filter and washed with high-purity water. After each cycle, both the adsorption capacity and the analytical blank were measured. These parameters remained stable for up to 12 cycles.

### 3.9. Analytical Figures of Merit

In this study, the limit of detection (LOD) and the limit of quantification were determined using the IUPAC definitions: LOD = 3 SD/m and LOD = 10 SD/m, where SD represents the standard deviation of 10 consecutive measurements of a blank solution, and m is the slope of the calibration curve [41]. The calculated limits of detection for Cr and V were 0.003 µg g^−1^ and 0.006 µg g^−1^, respectively. Additionally, the limits of quantification (LOQ) for Cr and V were found to be 0.01 µg g^−1^ and 0.02 µg g^−1^, respectively.

Five replicate measurements of the total procedure blank solution were conducted, and the relative standard deviation (RSD) of the background values for the raw data was calculated. The RSD ranged from 0.3% to 3.4% for Cr and from 0.9% to 4.6% for V. This indicates the imprecision of the total procedure. The calibration function exhibited a linear range of 1 to 50 µg L^−1^ for both elements.

The limit of detection achieved for Cr (0.05 µg L^−1^) is comparable with those obtained using magnetic ferrite [23], tailor-made polymer nanoparticles [25], and graphene oxide nanoparticles [26] as solid sorbents in dispersive micro-solid-phase extraction before determination by GF AAS. However, this limit is 17 times worse than the detection limit obtained with a graft copolymer made of polystyrene and polylinoleic acid combined with sodium iminodiacetate salt [24]. The limit of detection obtained for V (0.09 µg L^−1^) is twelve times better than that obtained using extraction induced by emulsion breaking [30] and comparable with limits of detection achieved using cloud point extraction based on the formation of a ternary complex between vanadium, 2-(2’-thiazolylazo)-p-cresol and ascorbic acid [27] and magnetic stirrer-induced dispersive ionic-liquid microextraction [29] for V preconcentration. However, this limit is nine times worse than the detection limit obtained using eutectic solvent extraction [28].

While directly comparing detection limits can be misleading due to the use of different systems, operating conditions, and modes, it is evident that the detection limit achieved using HR-CS GFAAS after adsorption onto exfoliated graphite is superior to that of conventional GFAAS. The preconcentration factors obtained for Cr and V were 28 and 34, respectively. The preconcentration factor is calculated using the ratio of the analyte concentration in the solid phase (C_1_) to the initial concentration of the analyte (C_0_) in the sample solution: PF = C_1_/C_0_ [42]. The liquid sample concentration used to assess detection limits and precision was 1 μg L^−1^.

### 3.10. Evaluation of the Environmental Impact

The environmental impact of the developed analytical procedure was assessed using the AGREEprep open-access software (version 0.91), which is based on the 12 principles of green analytical chemistry. The final score calculated for this procedure is 0.47. The results indicate that the environmental impact is average, primarily due to factors such as a lack of automation, insufficient miniaturisation, the use of non-bio-based reagents, as well as the volume of waste generated and the reliance on offline analysis.

### 3.11. Accuracy Verification

To ensure the accuracy and precision of the method, INCT-MP4-2 (Mixed Polish Herbs) certified reference material was analysed. This material was selected because it closely resembled the herbal samples being studied and was certified for the analytes of interest. The results for this certified reference material are summarised in Table 2. Short-term precision is reported as the standard deviation (SD) from five replicate measurements of each sample. The results align with the certified values for the reference material, as confirmed by a *t*-test at a 95% confidence level. This indicates that the proposed method is suitable for the preconcentration and determination of Cr and V in herbs after digestion and in herbal infusions.

### 3.12. Evaluation of Interferences

The reliability of the developed method was assessed by analysing a sample containing 50 µg L^−1^ of Cr and V. We examined the interference effects of coexisting ions such as potassium (K^+^), sodium (Na^+^), calcium (Ca^2+^), magnesium (Mg^2+^), zinc (Zn^2+^), aluminium (Al^3+^), and manganese (Mn^2+^) on the adsorption and determination of Cr and V under optimal conditions. The results indicate that, despite the presence of these coexisting ions, the recoveries of the analytes remained satisfactory. According to the data presented in Table 3, the recoveries for spiked samples ranged from 89% to 99% for Cr and from 87% to 98% for V. These findings suggest that the proposed method is effective for determining Cr and V in herbs and their infusions.

### 3.13. Standard Addition Tests

To confirm the accuracy of the proposed analytical method, standard addition tests were conducted on herbal samples spiked with Cr and V. The results presented in Table 4 are consistent with the added concentrations of Cr and V, as indicated by a *t*-test at a 95% confidence level. This suggests that the proposed method is effective for the determination of Cr and V in herbs and their infusions.

### 3.14. Determination of Cr and V in Herbs

To assess the usefulness of the proposed method for determining Cr and V content in herbal samples, the samples were analysed under the previously optimised experimental conditions. Three extraction procedures were conducted for each herb. The results for these analyses are presented in Table 5. The quantification of Cr and V was carried out using the standard calibration technique.

Based on the obtained results, it can be concluded that the best sources of Cr are motherwort, common milkweed, and horse chestnut bark. The extraction rates for these herbs vary from 40% to 60% and concentrations of Cr in infusions are the highest. The obtained extraction rates are consistent with reports found in the literature (40–70%) [43]. A high concentration of Cr was also determined in valerian roots after digestion; however, the extraction rate is only about 20% and the concentration of Cr in the infusion is significantly lower. The lowest concentrations of Cr were determined in hawthorn flower, hawthorn fruit, white mulberry leaf, and mistletoe. The concentration of Cr in these herbs after digestion is about two times lower than in motherwort, common milkweed, and horse chestnut bark. The extraction rates for these herbs are quite high and amount to 50%.

In contrast, there are no significant differences in vanadium concentration in samples after digestion depending on the herb type according to the ANOVA test. The exceptions are mistletoe and valerian roots, for which the concentration is lower. Also, vanadium extraction rates are notably higher for hawthorn flowers (76%) and white mulberry leaves (69%); however, for valerian root and horse chestnut bark, the rates are only about 20%. Similarly, the vanadium extraction rates align with the literature values, reported to be between 50% and 75% [43].

These findings suggest that the infusion preparation process is effective. However, it is important to note that the bioavailability of these elements in the body may be lower than the amounts present in the infusion. The results also suggest that infusions can be a good source of chromium and vanadium, particularly when certain types of herbs (e.g., motherwort and common milkweed) are used. Nonetheless, the efficiency of extraction varies depending on the herb type.

## 4. Conclusions

Based on the results obtained, it can be concluded that the proposed analytical method is suitable for the determination of Cr and V in herbs using HR-CS GFAAS after DMSPE, with exfoliated graphite as the solid sorbent.

This procedure also has several limitations. It cannot be used during chromium speciation analysis, and the elution of the analytes before HR-CS GFAAS determination is required due to the challenging process of slurry introduction into the atomiser. Additionally, some difficulties in maintaining slurry homogeneity can occur. As a result, the use of highly pure and expensive eluents is necessary. While this method is faster than classical solid-phase extraction, it remains time-consuming and laborious.

The study results indicate that certain herbs traditionally used to treat acquired cardiovascular diseases, diabetes, and obesity—such as hawthorn flower, hawthorn fruit, motherwort, white mulberry leaf, common milkweed, mistletoe, valerian root, and horse chestnut bark—are good sources of chromium and vanadium. Although these herbal infusions contain some beneficial elements, their low concentrations mean they cannot replace a balanced diet, which is essential for obtaining the necessary micronutrients. They should not completely replace modern medicine, but they can effectively complement it. It is also important to note that consuming these elements in amounts exceeding the recommended daily intake may be harmful to the human body.

## Figures and Tables

**Table 1:** Optimised experimental conditions for ultrasound-assisted dispersive micro-solid-phase extraction (UA DMSPE) using exfoliated graphite as a sorbent, coupled to HR-CS GFAAS for the determination of Cr and V.

UA DMSPE with Exfoliated Graphite		
Sample volume (mL)	10	
Amount of EGIC (mg)	5	
pH of the sample solution	6.5	
Ultrasonication time (s)	20 (10 for extraction and 10 for re-extraction)	
Centrifugation time (min)/rpm	3 (2 min before and 1 min after re-extraction)/4500	
Re-extraction solution/final vol. (μL)	2 mol L^−1^ HNO_3_/250	
**HR-CS GFAAS detection**		
	Cr	V
Wavelength (nm)	357.8687	318.3982
Lamp current (A)	9	
Spectral range (pixel)	200	
Dispersion (pm pixel^−1^)	2	
Read time (s)	5	
Delay time (s)	0	
Measurement mode	peak height	
Sample volume (μL)	20	
Modifier solution/vol. (μL)	---	3 mg mL^−1^ Mg(NO_3_)_2_/5
**Furnace programme steps**		
Drying	80 °C, ramp 6 °C s^−1^, hold 20 s	
Drying	90 °C, ramp 3 °C s^−1^, hold 20 s	
Drying	110 °C, ramp 5 °C s^−1^, hold 10 s	
Pyrolysis	350 °C, ramp 50 °C s^−1^, hold 20 s	
Pyrolysis	1400 °C, ramp 300 °C s^−1^, hold 10 s	1200 °C, ramp 300 °C s^−1^, hold 10 s
Atomisation	2400 °C, ramp 1500 °C s^−1^, hold 4 s	2700 °C, ramp 1500 °C s^−1^, hold 4 s
Cleanout	2450 °C, ramp 500 °C s^−1^, hold 4 s	2750 °C, ramp 500 °C s^−1^, hold 4 s

**Table 2:** Determination of Cr and V (concentration in mg kg^−1^) in INCT-MP4-2 (Mixed Polish Herbs) certified reference material using the HR-CS GFAAS technique after adsorption on exfoliated graphite.

Element	Certified Value	Found	Value of *t*-Test	Significance ^2^
Cr	1.69 ± 0.13	1.58 ± 0.11 ^1^	2.236	NS
V	0.952 ± 0.163	0.875 ± 0.094 ^1^	1.118	NS

^1^ standard deviation for five replicate measurements. ^2^ significance of *t*-test (n = 5) at 95% confidence level; *t*_critical_ = 2.776; NS: not significant.

**Table 3:** Effect of several foreign ions on the developed method for Cr and V determination.

Foreign Ion	Concentration of Foreign Ion (mg L^−1^)	Recovery ^a^ (%)	
		Cr	V
K^+^	20,000	93	87
Na^+^	20,000	96	89
Ca^2+^	10,000	91	92
Mg^2+^	3000	89	90
Al^3+^	600	95	94
Mn^2+^	200	98	96
Zn^2+^	30	99	98

^a^ recovery of a sample solution containing 50 µg L^−1^ of Cr and V. Percent recovery was calculated by the following equation: % recovery = (SSR SR)/SA∙100, where SSR—spiked sample result, SR—sample result, and SA—spike added.

**Table 4:** Determination of Cr and V (concentration in µg L^−1^) in herbal infusions following a standard addition using the HR-CS GFAAS technique after adsorption on exfoliated graphite.

Sample	Cr				V			
	Added	Found	Value of *t*-Test	Significance ^2^	Added	Found	Value of *t*-Test	Significance ^2^
Hawthorn flower	10	11.0 ± 1.1 ^1^	2.033	NS	10	10.5 ± 0.6 ^1^	1.863	NS
Hawthorn fruit	10	9.1 ± 0.8 ^1^	2.516	NS	10	10.2 ± 0.7 ^1^	2.555	NS
Motherwort	10	10.6 ± 0.8 ^1^	1.677	NS	10	11.1 ± 0.9 ^1^	2.733	NS
White mulberry leaf	10	10.9 ± 1.0 ^1^	2.012	NS	10	9.7 ± 0.7 ^1^	0.958	NS
Common milkweed	10	11.1 ± 1.0 ^1^	2.460	NS	10	10.4 ± 0.8 ^1^	1.118	NS
Mistletoe	10	9.6 ± 0.7 ^1^	1.278	NS	10	11.2 ± 1.1 ^1^	2.439	NS
Valerian root	10	9.2 ± 0.9 ^1^	1.988	NS	10	10.8 ± 0.9 ^1^	1.988	NS
Horse chestnut bark	10	10.3 ± 0.6 ^1^	1.118	NS	10	9.3 ± 0.8 ^1^	1.957	NS

^1^ standard deviation for five replicate measurements. ^2^ significance of *t*-test (n = 5) at 95% confidence level; *t*_critical_ = 2.776; NS: not significant.

**Table 5:** Determination of Cr and V (concentration in mg kg^−1^) in herbs after digestion and herbal infusions using the HR-CS GFAAS technique after adsorption on exfoliated graphite.

Sample	Cr		V	
	After Digestion	Herbal Infusion	After Digestion	Herbal Infusion
Hawthorn flower	0.99 ± 0.17 ^1^	0.54 ± 0.15 ^1^	0.227 ± 0.051 ^1^	0.172 ± 0.049 ^1^
Hawthorn fruit	0.84 ± 0.13 ^1^	0.34 ± 0.11 ^1^	0.236 ± 0.034 ^1^	0.062 ± 0.031 ^1^
Motherwort	2.03 ± 0.25 ^1^	1.10 ± 0.18 ^1^	0.256 ± 0.052 ^1^	0.152 ± 0.036 ^1^
White mulberry leaf	1.03 ± 0.22 ^1^	0.57 ± 0.16 ^1^	0.223 ± 0.19 ^1^	0.154 ± 0.035 ^1^
Common milkweed	1.72 ± 0.24 ^1^	1.03 ± 0.17 ^1^	0.248 ± 0.058 ^1^	0.149 ± 0.041 ^1^
Mistletoe	1.12 ± 0.27 ^1^	0.62 ± 0.18 ^1^	0.173 ± 0.044 ^1^	0.087 ± 0.034 ^1^
Valerian root	2.12 ± 0.31 ^1^	0.68 ± 0.19 ^1^	0.167 ± 0.042 ^1^	0.034 ± 0.008 ^1^
Horse chestnut bark	1.68 ± 0.25 ^1^	0.43 ± 0.12 ^1^	0.243 ± 0.058 ^1^	0.053 ± 0.017 ^1^

^1^ expanded uncertainty (for *p* = 95%, k = 2).

## Data Availability

The original contributions presented in this study are included in the article. Further inquiries can be directed to the corresponding author.
